# Short-Term Nicotine Improves Experimental Flap Survival: Implications for Different Findings in Experimental Flap Models

**DOI:** 10.3390/medicina62071398

**Published:** 2026-07-19

**Authors:** Gülce Sevdar Çeçen, Süleyman Çeçen, Özkan Yavaş, Nazım Haspolat, Sinan Çavun, Mine Sibel Gürün

**Affiliations:** 1 Department of Pharmacology, Faculty of Medicine, Bursa Uludag University, 16059 Bursa, Türkiye; nazimhaspolat@gmail.com (N.H.); scavun@uludag.edu.tr (S.Ç.); sgurun@uludag.edu.tr (M.S.G.); 2 Department of Plastic, Reconstructive and Aesthetic Surgery, Faculty of Medicine, Bursa Uludag University, 16059 Bursa, Türkiye; scecen@uludag.edu.tr; 3 Department of Pathology, Faculty of Veterinary Medicine, Bursa Uludag University, 16059 Bursa, Türkiye; oyavas@uludag.edu.tr

**Keywords:** nicotine, flap survival, ischemia–reperfusion injury, angiogenesis

## Abstract

*Background and Objectives*: The effects of nicotine on wound healing and flap survival remain controversial. While smoking is associated with increased flap complications, emerging evidence suggests that nicotine may exert dose- and duration-dependent effects. This study aimed to investigate how short-term, low-dose nicotine affects flap survival and to evaluate its potential role in oxidative stress and ferroptosis. *Materials and Methods*: Twelve adult male BALB/c mice were randomly assigned to either a control group or a nicotine-treated group, with six mice in each group. Nicotine, at a dose of 2 mg/kg, was given subcutaneously for fourteen days before flap surgery. This treatment continued for seven days after the surgery. A dorsal random-pattern skin flap model was established in all animals. The flap necrosis area was assessed on postoperative day 7 using planimetric analysis. Serum and tissue glutathione peroxidase 4 (GPX4) levels and serum malondialdehyde (MDA) levels were measured. Histopathological evaluation included epithelialization, microvascular density, neovascularization, fibroblast density, and inflammatory cell infiltration. *Results*: Nicotine treatment significantly reduced flap necrosis compared with the control group. Histopathological analysis demonstrated significantly increased epithelialization and microvascular density in the nicotine-treated group, whereas no significant differences were observed in neovascularization, fibroblast density, or inflammatory cell infiltration. Biochemical analyses revealed no significant differences in serum or tissue GPX4 levels or serum MDA levels between the groups. *Conclusions*: Short-term nicotine administration enhanced flap survival, correlating with increased epithelialization and microvascular density, while not significantly affecting markers of oxidative stress or ferroptosis. These results imply that the positive impact of nicotine on flap viability might be largely attributable to vascular processes rather than pathways involving oxidative stress.

## 1. Introduction

Flap surgeries are frequently used procedures in plastic and reconstructive surgery practice for replacing tissue lost due to trauma or burns, as well as for aesthetic purposes. However, despite advances in surgical techniques, tissue necrosis primarily associated with ischemia–reperfusion injury and the resulting decrease in flap survival leads to increased morbidity and prolonged hospital stays, significantly reducing surgical success [1].

Smoking is widely regarded as one of the major contributors to increased flap complication rates [2,3]. Studies have shown that not only smoking but also nicotine exposure increase the frequency of complications [4,5]. Experimental animal studies have also demonstrated that smoking or nicotine exposure impairs flap healing [6,7,8]. However, there are also studies showing that nicotine use impairs flap survival less than smoking [9].

Several studies have reported that nicotine administration increases neovascularization and vascular endothelial growth factor expression, thereby contributing to enhanced wound healing [10,11]. While the quantity of studies indicating enhanced wound or flap healing with nicotine is scarce, experimental investigations in cancer and atherosclerosis models have shown that nicotine can facilitate angiogenesis [12,13,14]. Furthermore, acute activation of α7-nicotinic acetylcholine receptors by nicotine has been shown to improve skin flap survival in rodents through the nitrergic system. This finding suggests that the effects of nicotine on wound healing and flap viability may be mediated through nicotinic receptor-dependent mechanisms [15].

In addition to its vascular effects, emerging evidence suggests that nicotine may potentially affect tissue injury through pathways associated with oxidative stress and ferroptosis. Ferroptosis is a type of controlled cell death characterized by iron-dependent lipid peroxidation and impaired antioxidant defense mechanisms, particularly involving glutathione peroxidase 4 (GPX4) [16,17]. Experimental studies have demonstrated that nicotine exposure can increase oxidative stress and lipid peroxidation markers such as malondialdehyde (MDA), therefore leading to ferroptotic cell damage in some tissues [18]. However, some studies have suggested that nicotine may also exert protective effects against ferroptosis under certain conditions. For instance, nicotine has been shown to reduce reactive oxygen species levels and increase GPX4 expression, thereby protecting cells from ferroptotic cell death in certain cancer models [19]. Taken together, these findings suggest that nicotine may exert a biphasic effect, whereby its impact on wound and flap healing may vary depending on the dose and duration of exposure.

The aim of the present study was to investigate the effects of short-term, low-dose nicotine administration on skin flap survival and to evaluate its association with oxidative stress- and ferroptosis-related markers. Given the conflicting findings reported in previous experimental studies, we sought to determine whether a short-term, low-dose nicotine exposure protocol might contribute to understanding the variability of nicotine-related outcomes observed in experimental flap models.

## 2. Materials and Methods

### 2.1. Animals and Experimental Design

Twelve adult male BALB/c mice weighing 20–25 g were included in this study. The animals were housed under standard laboratory conditions at a controlled temperature of 22 ± 2 °C with a 12 h light/12 h dark cycle (lights on at 07:00 a.m.), without reverse light exposure. Standard laboratory chow and water were provided ad libitum throughout the experimental period. All experimental procedures were approved by the local Institutional Animal Ethics Committee (Approval number 2024-04/03).

**Figure 1:**
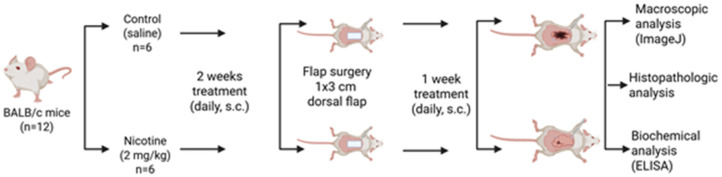
Schematic representation of the experimental design. Created with BioRender.com.

The animals were randomly assigned to either the control or nicotine-treated group (*n* = 6 per group, Figure 1). Nicotine hydrogen tartrate (2 mg/kg), dissolved in sterile 0.9% saline, was administered subcutaneously once daily for 14 consecutive days prior to flap surgery and continued for 7 days postoperatively. The control group received an equivalent volume of 0.9% saline according to the same schedule. The nicotine dose (2 mg/kg) used in this study was selected based on previous experimental studies showing that chronic administration of this dose for two weeks produces plasma nicotine levels comparable to those observed in habitual smokers [20]. The duration of nicotine administration was selected to represent a subchronic exposure model, allowing sufficient time for nicotine-induced vascular effects to develop prior to flap elevation while avoiding the excessive tissue damage associated with prolonged chronic exposure reported in previous studies [21,22,23]. Continued administration during the postoperative period was intended to maintain nicotine exposure throughout the critical phase of flap healing.

Following the two-week preoperative treatment period, a 1 × 3 cm^2^ random-pattern dorsal skin flap was elevated in all animals according to the McFarlane random-pattern dorsal skin flap model under sevoflurane anesthesia [24]. Anesthesia was induced with 3–5% sevoflurane and maintained at 1–3%, delivered in oxygen via a precision vaporizer. Postoperative treatments were continued for 7 days.

Predefined exclusion criteria included marked deterioration in general condition, reduced food intake, or body weight loss exceeding 10% during the study period. No animals met these criteria, and therefore, no animals were excluded from the analysis. The surgeon performing the flap procedures and the pathologist responsible for histopathological evaluation were blinded to group allocation.

### 2.2. Flap Viability and Necrotic Area Assessment

On postoperative day 7, the animals were euthanized, and the dorsal flaps were photographed using a standardized digital imaging system. The total flap area and necrotic regions were determined by macroscopic evaluation based on color, texture, and capillary refill characteristics. Planimetric analysis was performed using ImageJ software (Version 1.53, Bethesda, MD, USA), and necrotic areas were expressed as square centimeters (cm^2^).

### 2.3. Tissue and Serum Biochemical Analyses

At the end of the experimental period, blood samples were collected from the subclavian vein under terminal anesthesia and centrifuged at 3000 rpm for 10 min to obtain serum. The serum samples were stored at −80 °C until analysis.

Flap tissue samples were harvested and immediately washed with ice-cold phosphate-buffered saline (PBS) to remove residual blood. The tissues were then homogenized in cold phosphate-buffered saline using a mechanical homogenizer (Heidolph DIAX900, Schwabach, Germany) and subsequently centrifuged to obtain clear supernatants for biochemical analyses.

GPX4 levels in both tissue homogenates and serum samples were quantified using commercially available enzyme-linked immunosorbent assay (ELISA) kits according to the manufacturer’s instructions (BT Laboratory, China; Cat. No. E2344Mo). MDA levels were also determined using a commercially available ELISA kit (Shanghai YL Biotech, Shanghai, China; Cat. No. YLA0319MO).

The results obtained from tissue samples were normalized to total protein content and expressed as concentration per milligram of protein.

### 2.4. Histopathological Examination

Flap tissue samples were fixed in 4% paraformaldehyde at +4 °C for 48 h and processed through routine dehydration and clearing steps before paraffin embedding. Paraffin blocks were sectioned at 4 µm thickness using a microtome (Leica Microsystems, Wetzlar, Germany), mounted on glass slides, and stained with hematoxylin and eosin (H&E) following standard protocols [25].

Histopathological evaluation was performed under a light microscope by an experienced observer blinded to the experimental groups. For each slide, five non-overlapping representative fields from the central portion of the flap were evaluated. General tissue architecture, epithelialization, and neovascularization were assessed at 100× magnification, whereas fibroblast density, neutrophil infiltration, epithelial thickness, and microvascular density were evaluated at 400× magnification.

Neovascularization, fibroblast density, epithelialization, epithelial thickness, and neutrophil infiltration were semi-quantitatively scored on a 0–3 scale: 0 = none/absent, 1 = mild, 2 = moderate, and 3 = severe/marked. Microvascular density was scored separately on a 0–4 scale, with higher scores indicating a greater number of microvessels in the evaluated fields, as previously described [26,27]. The histopathological scores were analyzed and reported separately for each parameter. The scoring system was adapted from previously published histological wound-healing and tissue-repair assessment methods and was not specifically validated for the present flap model; therefore, these findings were interpreted as supportive semi-quantitative data.

### 2.5. Sample Size Justification and Post Hoc Power Analysis

Group size was determined in accordance with previous experimental studies employing similar flap models and in line with ethical considerations to minimize animal use. After completion of the study, a post hoc power analysis was performed based on the primary outcome (flap necrosis area). Using the observed group means (control: 1.049 ± 0.4071; nicotine: 0.2870 ± 0.1817; *n* = 6 per group), the standardized effect size was calculated as Cohen’s d = 2.42. Assuming a two-sided α level of 0.05, the statistical power achieved was estimated to be approximately 96%, indicating that the sample size was sufficient to detect the observed difference between groups.

### 2.6. Statistical Analyses

Statistical analyses were performed using GraphPad Prism software (version 11.0.0, GraphPad Software, San Diego, CA, USA). Quantitative data were expressed as mean ± standard error of the mean (SEM), while semi-quantitative data were presented as the median (interquartile range, IQR). Normality of data distribution was assessed using the Shapiro–Wilk test. For comparisons between two groups, either an independent samples *t*-test or the Mann–Whitney U test was used as appropriate. A *p*-value < 0.05 was considered statistically significant.

## 3. Results

### 3.1. Effect of Nicotine on Flap Viability and Necrotic Area

**Figure 2:**
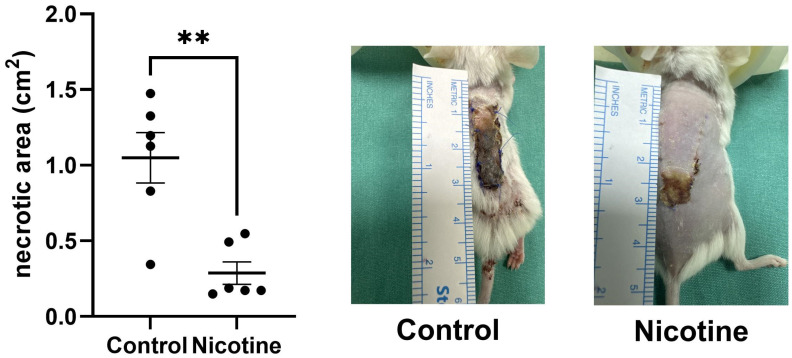
Effect of nicotine on flap necrosis area. Necrotic area of dorsal skin flaps in control and nicotine-treated groups on postoperative day 7. Data are presented as mean ± SEM. Individual data points are shown. Statistical analysis was performed using the Mann–Whitney U test. ** *p* < 0.01.

The percentage of necrotic areas was significantly lower in the nicotine-treated group compared with the control group. The mean necrotic area was 1.049 ± 0.1662 cm^2^ in the control group and 0.2870 ± 0.07417 cm^2^ in the nicotine-treated group. Statistical analysis using the Mann–Whitney U test revealed a significant difference between the groups (*p* = 0.0087). These findings indicate a marked reduction in flap necrosis in animals receiving nicotine treatment (Figure 2).

### 3.2. Histopathological Findings

**Figure 3:**
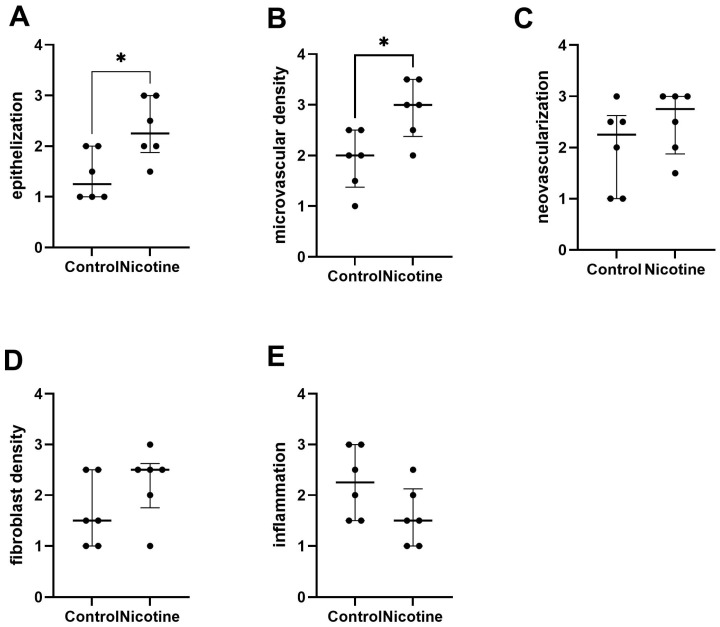
Histopathological evaluation of flap tissues in control and nicotine-treated groups. (**A**) Epithelialization scores, (**B**) microvascular density, (**C**) neovascularization, (**D**) fibroblast density, and (**E**) inflammatory cell infiltration in control and nicotine-treated groups. Data are presented as median with interquartile range (IQR). Individual data points are shown for each group. * *p* < 0.05.

Histopathological data were analyzed using the Mann–Whitney U test. Histopathological analysis demonstrated a significant increase in epithelialization scores in the nicotine-treated group compared with the control group (*p* = 0.0368, Figure 3A), with median values of 1 (IQR: 1–2) in the control group and 2 (IQR: 2–3) in the nicotine-treated group. Similarly, microvascular density was significantly higher in the nicotine-treated group (*p* = 0.0303, Figure 3B), with median scores of 2 (IQR: 1–3) in the control group and 3 (IQR: 2–4) in the nicotine-treated group.

In contrast, no significant differences were observed between the groups in neovascularization (*p* = 0.3009, Figure 3C) or fibroblast density (*p* = 0.2100, Figure 3D), although higher median values were noted in the nicotine-treated group for both parameters. The median neovascularization scores were 2 (IQR: 1–3) and 3 (IQR: 2–3), and fibroblast density scores were 2 (IQR: 1–3) and 3 (IQR: 2–3) in the control and nicotine-treated groups, respectively.

**Figure 4:**
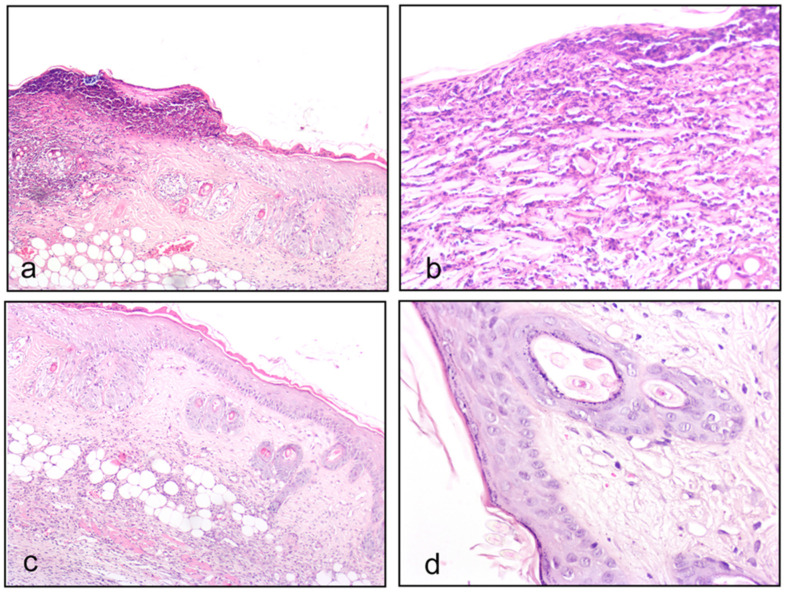
Histological appearances of skin tissues from the experimental groups stained with hematoxylin and eosin (H&E). (**a**,**b**) Sections from the control group showing impaired wound healing, characterized by disrupted or absent epithelialization, prominent inflammatory cell infiltration, bacterial colonies, and areas of hyperemia and hemorrhage in the hypodermis (10× and 40×). (**c**,**d**) Sections from the nicotine-treated group demonstrating increased epithelialization and regenerative features, along with the presence of capillary vessels and reduced inflammatory cell infiltration (10× and 40×).

Inflammatory cell infiltration was lower in the nicotine-treated group; however, this difference did not reach statistical significance (*p* = 0.1364, Figure 3E), with median scores of 2 (IQR: 1–3) in the control group and 2 (IQR: 1–2) in the nicotine-treated group. Representative histopathological images of the study groups are presented in Figure 4.

### 3.3. Effect of Nicotine on Biochemical Analyses

#### 3.3.1. Serum and Tissue GPX4 Levels

**Figure 5:**
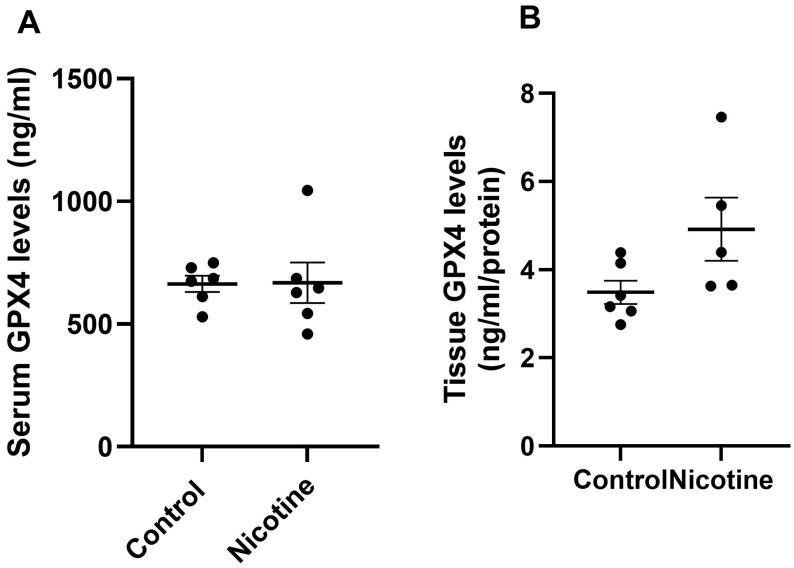
Effects of nicotine on GPX4 levels. (**A**) Serum GPX4 levels and (**B**) tissue GPX4 levels in control and nicotine-treated groups. No significant differences were observed between the groups. Data are presented as mean ± SEM. Individual data points are shown.

There was no significant difference in serum GPX4 levels between the control and nicotine-treated groups (*p* = 0.96, Figure 5A). The mean serum GPX4 level was 663.5 ± 33.22 in the control group and 668.0 ± 82.2 in the nicotine-treated group.

Similarly, no significant difference was observed in tissue GPX4 levels between the groups (*p* = 0.12, Figure 5B). The mean tissue GPX4 level was 3.489 ± 0.26 in the control group and 4.92 ± 0.72 in the nicotine-treated group (*n* = 6 for the control group and *n* = 5 for the nicotine group). One tissue sample from the nicotine-treated group exceeded the upper quantification limit of the ELISA and was therefore not included in the tissue GPX4 analysis.

#### 3.3.2. Serum MDA Levels

**Figure 6:**
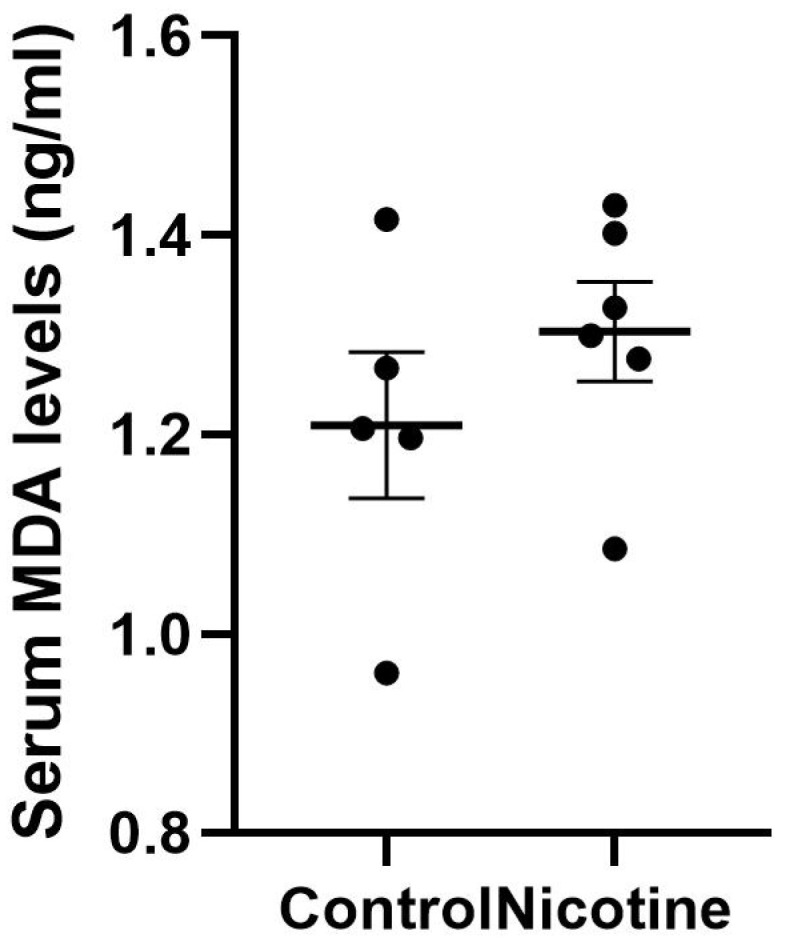
Serum MDA levels. Serum MDA levels in control and nicotine-treated groups. No significant difference was observed between the groups. Data are presented as mean ± SEM. Individual data points are shown.

There was no significant difference between serum levels of MDA for the groups (*p* = 0.32, Figure 6). The mean serum level of the control group was 1.209 ± 0.07 and 1.30 ± 0.05 in the nicotine-treated group.

## 4. Discussion

The present study investigated the effects of short-term nicotine administration on skin flap survival and its potential association with oxidative stress and ferroptosis-related pathways. The main findings of this study were that short-term nicotine administration reduced flap necrosis and improved selected histopathological indicators of flap healing, while no significant alterations were detected in the oxidative stress- and ferroptosis-related markers assessed. These findings indicate that nicotine may exert beneficial effects on flap viability.

The effects of nicotine on flap survival remain controversial in the literature. While smoking is widely associated with increased flap complications, several clinical and experimental studies have suggested that nicotine alone may exert different biological effects depending on dose and duration of exposure [12,15,28,29]. The reduced necrotic area observed in the nicotine-treated group in the present study supports the hypothesis that nicotine may have beneficial effects on tissue viability when administered for a limited duration.

One possible explanation for this finding is the ability of nicotine to influence microvascular perfusion and angiogenic signaling [12,30]. Previous experimental studies have demonstrated that activation of nicotinic acetylcholine receptors may enhance endothelial cell activity and promote angiogenesis [15]. Improved microvascular circulation may therefore contribute to the reduced necrosis observed in the nicotine-treated group.

Histopathological evaluation further supported the beneficial effects of short-term nicotine on flap healing. In the present study, epithelialization and microvascular density significantly increased in the nicotine-treated group, suggesting enhanced tissue repair and microvascular remodeling. These findings are consistent with previous experimental studies reporting that nicotine can promote epithelialization and angiogenesis, thereby contributing to improved wound healing [10,11]. Although neovascularization and fibroblast density were higher in the nicotine-treated group, these differences did not reach statistical significance, possibly due to the limited sample size or the semi-quantitative nature of the scoring system. In addition, inflammatory cell infiltration tended to be lower in the nicotine-treated group, indicating a potential modulatory effect of nicotine on the inflammatory response, although this finding was not statistically significant. Taken together, these findings suggest that short-term, relatively low-dose nicotine may improve flap healing by promoting microvascular adaptation and epithelial regeneration rather than by markedly altering fibroblast proliferation or inflammatory cell infiltration. This pattern is consistent with previous experimental evidence indicating that nicotine can influence endothelial cell activity and microcirculation, thereby contributing to improved tissue viability.

In the present study, biochemical analyses revealed no significant differences in serum MDA levels or GPX4 levels in serum and tissue samples between the groups. These findings suggest that the observed improvement in flap survival may not be primarily mediated through systemic changes in oxidative stress or ferroptosis-related pathways. Instead, the beneficial effect of nicotine observed in this model may be more closely related to its vascular or microcirculatory effects. Indeed, previous studies have reported that nicotine may increase MDA levels and promote ferroptosis under certain experimental conditions, indicating that its effects on oxidative stress pathways may vary depending on dose and duration of exposure [31,32,33,34].

It should be noted that serum MDA and GPX4 measurements were primarily included to assess systemic oxidative stress and antioxidant responses following systemic nicotine administration. However, ferroptosis is predominantly a tissue-level process, and circulating biomarkers may not fully reflect local ferroptotic activity within the flap tissue. Although tissue GPX4 levels were evaluated to provide a more localized assessment of a key ferroptosis-related pathway, tissue MDA levels were not measured. Consequently, local lipid peroxidation within the flap tissue may not have been fully characterized, representing a limitation of the present study. In addition, other established markers of ferroptosis, such as tissue-specific lipid peroxidation markers, iron accumulation, and other ferroptosis-pathway components, were not evaluated. Therefore, the present findings should not be interpreted as excluding a role for ferroptosis in flap survival, but rather as indicating that no alterations were detected in the ferroptosis-related markers assessed in this study.

Experimental studies investigating the effects of nicotine on flap survival have reported heterogeneous findings, likely due to differences in dosing regimens, duration of exposure, and nicotine formulations. Several studies employing chronic nicotine administration protocols (e.g., 2–4 mg/kg for 20–47 days) have demonstrated either increased flap necrosis or no significant difference compared with controls [21,22,23,35,36]. For instance, prolonged nicotine exposure for 28–47 days has been associated with impaired flap survival or unchanged necrosis rates, even at similar doses [21,23,35]. Similarly, repeated nicotine administration at 2 mg/kg, including twice-daily protocols, has been shown to increase necrosis [22,36]. In contrast, studies investigating acute or short-term nicotine exposure have demonstrated dose-dependent effects, with lower doses (up to 2 mg/kg) showing potential protective effects on flap survival [15]. An important consideration when interpreting these findings is that only a single nicotine dose and exposure duration were evaluated in the present study. Although our results are consistent with the concept of biphasic nicotine effects, the current design does not permit definitive conclusions regarding dose–response or time-dependent relationships. Therefore, when nicotine is used to model cigarette smoking in experimental flap studies, careful consideration should be given to the selected dosing regimen, exposure duration and nicotine formulation.

Importantly, many of these studies do not clearly specify the chemical form of nicotine used (e.g., free base versus salt forms such as nicotine hydrogen tartrate), which may influence pharmacokinetics and biological responses. Therefore, variability in nicotine formulation, in addition to dose and duration, should be considered when interpreting these findings.

In this context, the present study utilized a relatively low-dose, short-term nicotine administration protocol, which may partly explain the improved flap viability observed compared with previous chronic exposure models.

Although the present findings demonstrate improved flap viability under the specific experimental conditions evaluated, their direct clinical applicability remains uncertain. Important differences between experimental models and clinical settings, including species-specific responses, nicotine pharmacokinetics, exposure patterns, comorbidities, and surgical variables, may substantially influence outcomes. Therefore, the current results should primarily be interpreted as contributing to the understanding of nicotine’s biological effects in experimental flap models and as a basis for future translational investigations rather than as evidence supporting a specific clinical application of nicotine. Importantly, the present findings should not be interpreted as contradicting the well-established adverse effects of cigarette smoking on flap outcomes. Cigarette smoke contains numerous toxic compounds in addition to nicotine, including carbon monoxide, oxidants, and other substances that may impair tissue perfusion and wound healing. Furthermore, multiple patient- and procedure-related factors influence clinical flap complications. Therefore, short-term nicotine administration in a controlled experimental setting may not fully reflect the complex biological consequences of tobacco use in clinical practice.

This study has several limitations. First, the relatively small sample size may have influenced the statistical significance of secondary outcomes, particularly semi-quantitative histopathological parameters. Although post hoc power analysis demonstrated adequate power for the primary outcome (flap necrosis area), the study was not specifically powered to detect differences across all secondary endpoints. Second, only a single nicotine dose and exposure duration were evaluated; therefore, dose–response relationships and the effects of different exposure protocols could not be assessed. This limitation is particularly important given the possibility that nicotine may exert dose- and duration-dependent biological effects and prevents definitive conclusions regarding the influence of dose and duration on flap viability. Third, although serum MDA and GPX4 levels and tissue GPX4 levels were evaluated, tissue MDA levels were not measured. As ferroptosis is primarily a tissue-level process, local lipid peroxidation within the flap tissue may not have been fully characterized. Furthermore, direct assessment of flap perfusion was not performed. Therefore, although increased microvascular density suggests a possible vascular contribution, the role of perfusion-related mechanisms could not be directly confirmed. In addition, the findings are based on a single experimental flap model, which may limit their generalizability across different flap types or experimental conditions. Future studies employing multiple doses, exposure durations, experimental models, direct perfusion assessments, and additional tissue-specific ferroptosis markers are needed to better characterize the therapeutic and adverse effects of nicotine and to further elucidate its underlying mechanisms of action.

## 5. Conclusions

Short-term nicotine administration improved flap survival, as evidenced by reduced necrosis and enhanced epithelialization and microvascular density. No significant changes were observed in the oxidative stress- and ferroptosis-related markers assessed in the present study, suggesting a possible contribution of vascular and microcirculatory mechanisms. These findings contribute to the understanding of variable effects of nicotine as reported in experimental flap studies and emphasize the importance of careful consideration of dose, duration and exposure conditions when interpreting experimental outcomes.

## Data Availability

The data supporting the findings of this study are available from the corresponding author upon reasonable request.

## Abbreviations

The following abbreviations are used in this manuscript:

MDA	Malondialdehyde
GPX4	Glutathione Peroxidase 4
ELISA	Enzyme-Linked Immunosorbent Assay
H&E	Hematoxylin and Eosin
SEM	Standard Error of the Mean
IQR	Interquartile Range
